# Exploring the predictive power of jejunal microbiome composition in clinical and subclinical necrotic enteritis caused by *Clostridium perfringens*: insights from a broiler chicken model

**DOI:** 10.1186/s12967-023-04728-w

**Published:** 2024-01-19

**Authors:** Hemlata Gautam, Lisanework E. Ayalew, Noor Ahmad Shaik, Iresha Subhasinghe, Shelly Popowich, Betty Chow-Lockerbie, Alexa Dixon, Khawaja Ashfaque Ahmed, Suresh K. Tikoo, Susantha Gomis

**Affiliations:** 1 Department of Veterinary Pathology, Western College of Veterinary Medicine, University of Saskatchewan, 52 Campus Drive, Saskatoon, SK S7N 5B4 Canada; 2 Vaccinology and Immunotherapy, School of Public Health, University of Saskatchewan, 5D40 Health Sciences, 107 Wiggins Road, Saskatoon, SK S7N 5E5, Canada

**Keywords:** Necrotic enteritis, Broiler chickens, Human, Microbiome dysbiosis, Toxin gene virulence

## Abstract

**Background:**

Necrotic enteritis (NE) is a severe intestinal infection that affects both humans and poultry. It is caused by the bacterium *Clostridium perfringens* (CP), but the precise mechanisms underlying the disease pathogenesis remain elusive. This study aims to develop an NE broiler chicken model, explore the impact of the microbiome on NE pathogenesis, and study the virulence of CP isolates with different toxin gene combinations.

**Methods:**

This study established an animal disease model for NE in broiler chickens. The methodology encompassed inducing abrupt protein changes and immunosuppression in the first experiment, and in the second, challenging chickens with CP isolates containing various toxin genes. NE was evaluated through gross and histopathological scoring of the jejunum. Subsequently, jejunal contents were collected from these birds for microbiome analysis via 16S rRNA amplicon sequencing, followed by sequence analysis to investigate microbial diversity and abundance, employing different bioinformatic approaches.

**Results:**

Our findings reveal that CP infection, combined with an abrupt increase in dietary protein concentration and/or infection with the immunosuppressive variant infectious bursal disease virus (vIBDV), predisposed birds to NE development. We observed a significant decrease (p < 0.0001) in the abundance of *Lactobacillus* and *Romboutsia* genera in the jejunum, accompanied by a notable increase (p < 0.0001) in *Clostridium* and *Escherichia*. Jejunal microbial dysbiosis and severe NE lesions were particularly evident in birds infected with CP isolates containing *cpa*, *netB*, *tpeL*, and *cpb2* toxin genes, compared to CP isolates with other toxin gene combinations. Notably, birds that did not develop clinical or subclinical NE following CP infection exhibited a significantly higher (p < 0.0001) level of *Romboutsia*. These findings shed light on the complex interplay between CP infection, the gut microbiome, and NE pathogenesis in broiler chickens.

**Conclusion:**

Our study establishes that dysbiosis within the jejunal microbiome serves as a reliable biomarker for detecting subclinical and clinical NE in broiler chicken models. Additionally, we identify the potential of the genera *Romboutsia* and *Lactobacillus* as promising candidates for probiotic development, offering effective alternatives to antibiotics in NE prevention and control.

**Supplementary Information:**

The online version contains supplementary material available at 10.1186/s12967-023-04728-w.

## Introduction

Necrotic enteritis (NE) is a severe inflammatory infection that affects the small intestine, specifically the jejunum and ileum. This condition can occur in both humans and poultry. The causative agent of NE is *Clostridium perfringens* (CP), a gram-positive, rod-shaped, spore-forming bacterium that thrives in various environments like soil, litter, and dust. In chickens, CP can act as an opportunistic pathogen. However, in humans, the primary mode of transmission is through the consumption of contaminated food, including undercooked meat or poultry [1]. CP is divided into seven groups (A to G) based on major toxin producing genes including alpha (*cpa*), beta (*cpb*), epsilon (*etx*), iota (*itx*), enterotoxin (*cpe*) and necrotic enteritis B like toxin (*netB*) [2]. Affected broiler chickens develop NE between 2 and 6 weeks of age [3]. The disease has clinical and subclinical forms; the clinical form includes depression, diarrhea, dehydration, ruffled feathers, low body weight and increased feed conversion ratio (FCR) [4]. Mortality can reach up to 1% per day with a total mortality of 10–40% [5]. The subclinical form of NE is the most common in the field and results in an increased feed conversion ratio (FCR) and reduction in weight gain which leads to overall poor performance and economic losses [6, 7]. Economic losses are expected to rise as many countries as possible [8, 9], including Canada are voluntarily reducing and withdrawing the prophylactic use of antibiotics. Since there is no effective control available against NE, development of alternative prophylactics and therapeutic agents is of utmost importance. In this context, chickens serve as valuable animal models for studying NE as they share some similarities with humans in terms of disease manifestation [10–12], host-pathogen interaction [13], genetics [14], intestinal physiology [15], and experimental feasibility[16], thereby facilitating the investigation of potential therapeutic interventions.

The pathogenesis of CP is poorly understood. However, several predisposing factors such as infections with coccidia, immunosuppressive viruses, and abrupt change in protein contents of the diet have been identified as contributing factors for the adhesion and colonization of the bacteria in the intestinal mucosa. In addition, *cpa,* [17], *netB* [18] and *tpeL* [19, 20] toxins play important roles in the pathogenesis of NE. Of these, *cpa* mainly disrupts the membrane of enterocytes by hydrolysing phosphatidylcholine and sphingomyelin, producing discylglycerol and ceramide respectively [21, 22]. Furthermore, *cpa* activates the arachidonic acid pathway inducing the production of prostaglandins, tromboxanes, leukotriens and lactate dehydrogenase and result in necrosis of the intestine [23]. *NetB* is a pore forming toxin [24] and permits an influx of Na^+^, Cl^-^ and Ca^+2^ into the cytoplasm and causes osmotic lysis of the cell [25, 26]. Most clinical isolates of CP produce *netB* [27]. *NetB* mutants were unable to produce NE in broiler chickens [18]. The co-expression of *tpeL* and *netB* by CP can lead to a rapid onset of NE with higher mortality [19, 20]. *TpeL* induces host cell apoptosis by inhibiting the Ras signalling pathway [28, 29].

The chicken intestinal microbiota plays a crucial role in the growth and health of chickens, contributing towards improved nutrient absorption, improved immune system and inhibition of pathogen colonization by competitive exclusion [30]. Multiple studies demonstrated that *Eimeria* sp. induced alteration of the cecal microbiome can predispose chickens to the development of NE [31, 32]. However, the impact of the change in the microbial community in the pathogenesis of NE is not clearly understood [32, 33]. The objectives of this study were to; (a) develop subclinical and/or clinical NE broiler chicken animal model by inducing immunosuppression using infectious bursal disease virus (IBDV) and/or by abrupt increase of the protein content in the chicken feed, (b) investigate the impact of the intestinal microbiome in the pathogenesis of NE, and (c) study the virulence of CP isolates containing different combinations (*cpa*, *netB*, *tpeL* and/or *cpb2*) of toxin genes including their impact on the jejunal microbiome.

## Results

**Fig. 1:**
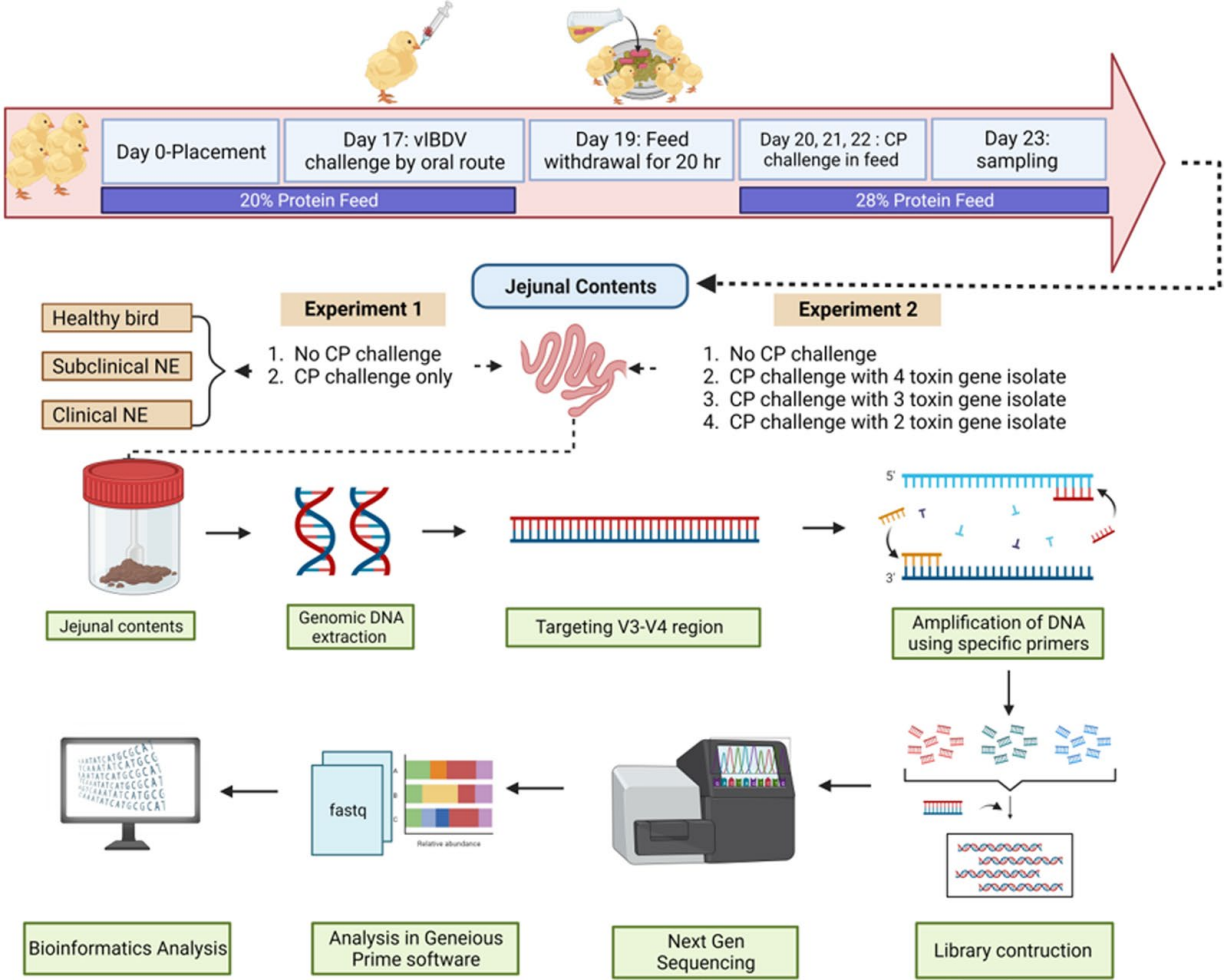
Experimental design and sample processing for *Clostridium perfringens* (CP) animal challenge. This figure provides a diagrammatic representation of the CP animal challenge conducted during both experiments 1 and 2. It delineates the timeline for sample collection and the subsequent processing steps essential for Illumina MiSeq-based next-generation sequencing, targeting the 16S rRNA gene’s V3–V4 region. Image “created with www.BioRender.com” accessed on October 22, 2023

The experimental procedures undertaken in this study are outlined in a schematic representation featured in Fig. 1.

### Toxin gene makeup of CP isolates

Among the 57 CP isolates, 44 and 12 isolates were classified as toxinotype G and A, respectively. However, one isolate contained *cpa*+*netB*+*cpiap*+cpb2 and did not fit into the current CP toxinotype classification. Overall, 7 toxin gene combinations were found among the analyzed CP clinical isolates including *cpa*+*netB*+*cpb2*(54.4%), *cpa*+*netB* (12.28%), *cpa*+*netB*+*cpb2*+*tpeL* (10.5%), *cpa*+*cpb2* (8.77%), *cpa* alone (10.5%), *cpa*+*netB*+*cpiap*+*cpb2* (1.7%), and *cpa*+*cpb2*+*tpeL* (1.7%)**.** None of these isolates harbored *cpe*, *cpb* or *etx* toxin genes.

### Development of a NE animal model

#### Clinical signs and mortality

**Fig. 2:**
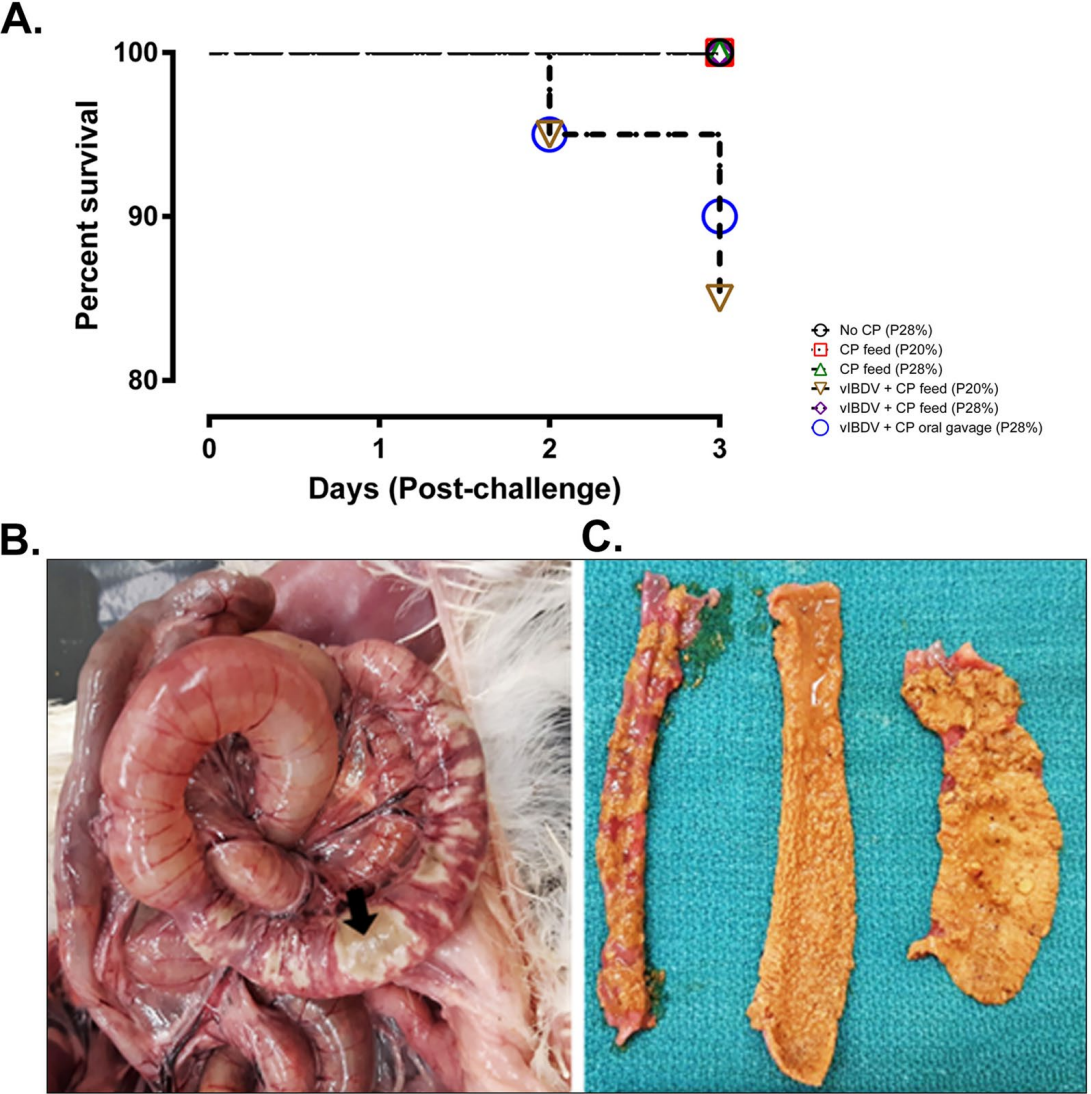
Survival and macroscopic NE lesions of groups of broiler chickens (n = 20/group) post-CP challenge. **A** Highlights a mortality rate ranging between 10 to 15% in groups of birds pre-exposed to vIBDV SK09 before the CP challenge (p = 0.0173). Key terms: CP: *Clostridium perfringens*; P: protein; vIBDV: variant infectious bursal disease virus. **B** Displays a dilated jejunum with patchy, thin, necrotizing areas (arrow) of the intestinal mucosa through the serosal surface of the intestine. **C** Depicts severe, diffuse, acute necrosis of the intestinal mucosa in the mucosal surface of the intestine

In the developed animal infection model, a total mortality of 10% and 15% (p=0.0173) was observed in the group with vIBDV SK09 + CP by oral gavage (28% protein) and vIBDV SK09 + CP in feed (20% protein) group, respectively (Fig 2A). Death was peracute after the development of clinical signs due to toxemia. The rest of the infected birds were apparently normal until the end of the clinical trial and the only clinical sign observed was diarrhea. At 2 days post-infection (DPI), one bird was found dead in both the vIBDV SK09 + CP by oral gavage (28% protein) group and vIBDV SK09 + CP in feed (20% protein) group. At 3 DPI, one and two birds were found dead in the vIBDV SK09 + CP by oral gavage (28% protein) and vIBDV SK09 + CP in feed (20% protein) groups, respectively (Fig 2A). No mortality was observed in the other experimental groups throughout the study period.

#### Gross and microscopic lesions

**Fig. 3:**
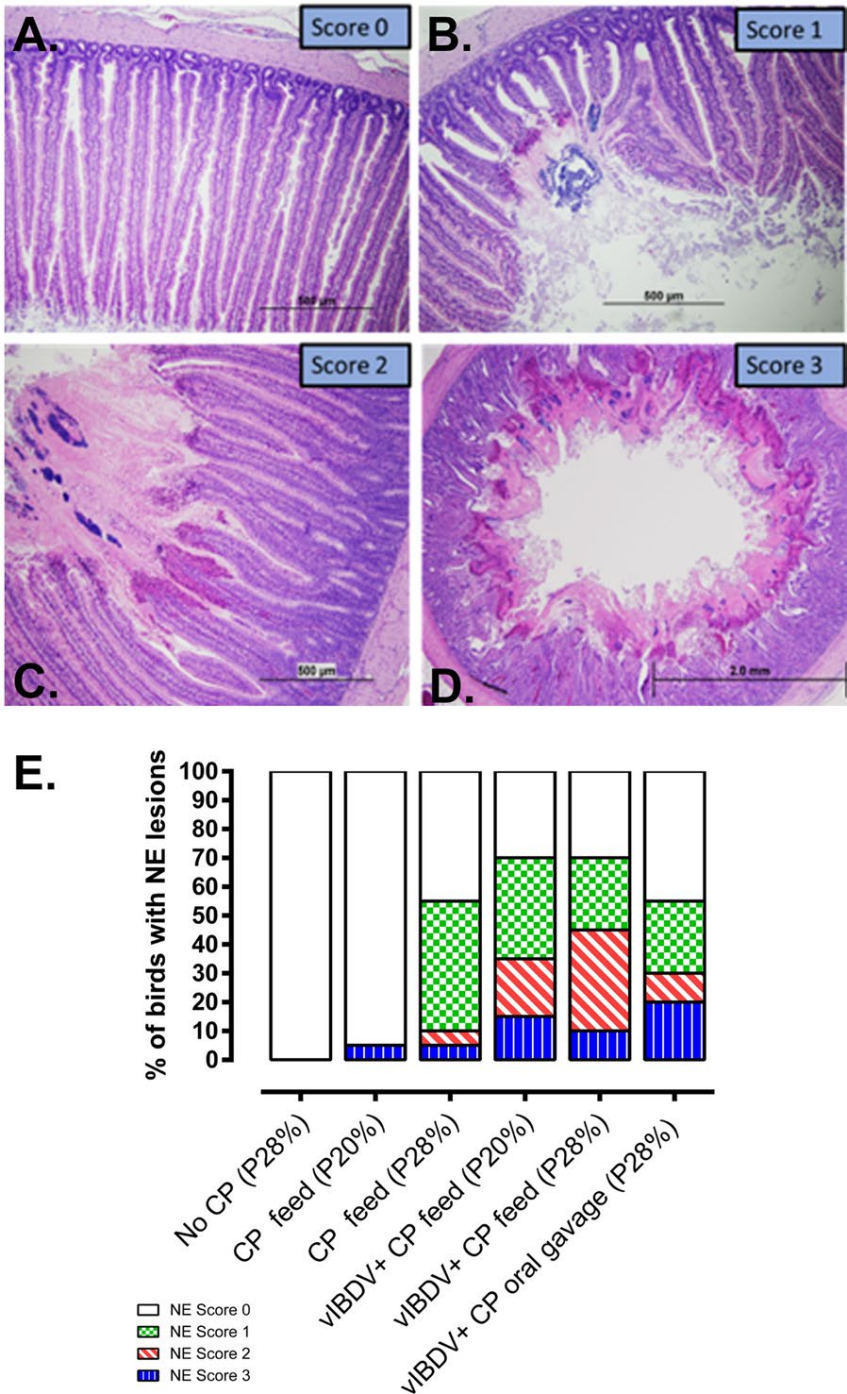
Histopathological analysis and scoring of jejunal NE lesions in broiler chickens (n = 20/group). **A** Represents the normal intestinal mucosa (0 score), **B** shows mild, focal, acute necrosis of intestinal villi (1 score), **C** shows moderate, multifocal to coalescing, acute necrosis of intestinal villi (2 score) and **D** represents severe, diffuse, acute necrosis of intestinal villi (3 score). **E** percentage microscopic NE lesions in birds (n = 20/group) which were predisposed to abrupt change in protein content in the diet and/or infection with vIBDV followed by exposure to CP (p < 0.0001). (CP: *Clostridium. perfringens*; P20: 20% protein in the feed; P28: 28% protein in the feed; vIBDV: variant infectious bursal disease virus)

On gross examination, intestines were dilated with gas and patchy necrotic areas were visible through the serosal surface (Fig 2B). Upon opening the intestines, severe, diffuse necrosis was evident in the duodenum, jejunum, and the ileum in the dead or euthanized birds (Fig 2C). Gross NE lesions were observed at a frequency of 5%, 5%, 15%, 10% and 20% in Groups 2 (CP feed, 20% protein), 3 (CP feed, 28% protein), 4 (vIBDV + CP feed [20% protein]), 5 (vIBDV + CP feed [28% protein]) and 6 (vIBDV + CP oral gavage [28% protein]), respectively. No gross lesions were observed in the unchallenged control group. In this NE model, intestinal necrosis was pronounced and prevalent in the jejunum compared to the duodenum or ileum. The histopathological lesions were scored from 0 to 3 (Fig 3A–D). Some of the CP exposed birds had extensive, severe, diffuse necrosis of intestinal villi with the presence of occasional bacterial colonies (Fig 3D) with few birds harboring fibrinonecrotic debris in the lumen. Groups 2, 3, 4, 5 and 6 had histopathological lesions of NE at a frequency of 5%, 55%, 70%, 70% and 55%, respectively (Fig 3E, p < 0.0001). No histologic lesions were observed in the unchallenged negative control group.

#### 16S rRNA microbiome sequence summary

Next generation 16S rRNA gene sequencing-based microbiome analysis was performed to examine the pattern of dysbiosis (i.e., a shift in the composition and relative abundance of the jejunal microbiome of the intestinal microbial community in CP infected birds as compared to the non-infected control group with healthy intestine). The average number of raw sequences read obtained per sample was 914,290 and it was consistent within and between the groups. After trimming/filtering and removing reads with quality scores of less than 30, an average of 679,150 reads per sample was obtained. The average length of the assembled reads used for taxonomic classification was 465 base pairs (bp). All the FastQ files are deposited in the GenBank with Bio project numbers PRJNA1023687 and PRJNA1024573.

#### Jejunal relative microbial abundance at the phylum level

**Fig. 4:**
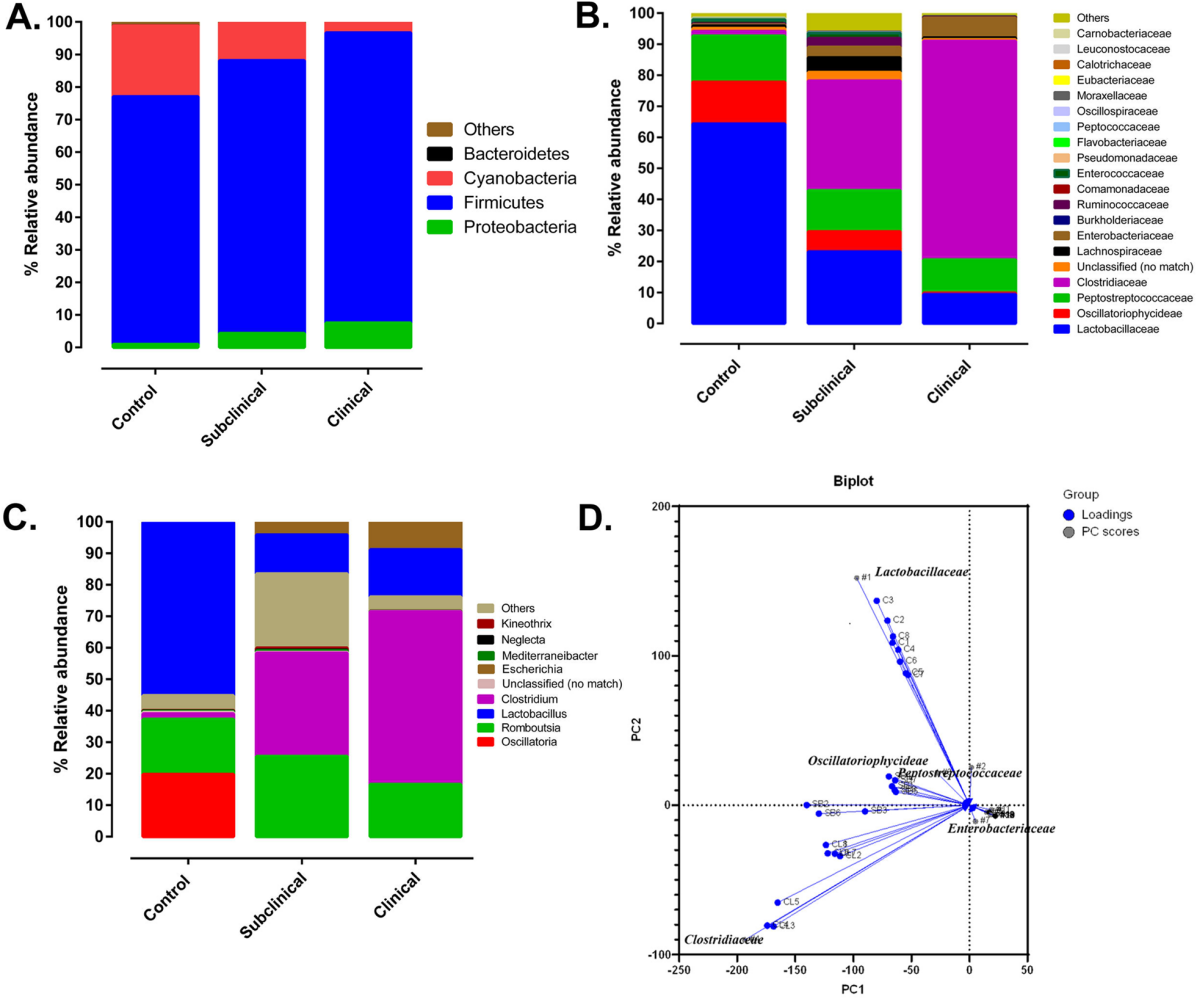
Analysis of jejunal microbiome in broiler chickens with subclinical or clinical NE based on 16S amplicon sequencing.** A** Depicts the relative abundance distribution at the phylum level. **B** Illustrates the relative abundance distribution at the family level. **C** Demonstrates the relative abundance distribution at the genus level. **D** Presents a principal component analysis biplot (n = 8/group) that visually captures the distances and relationships among three distinct groups: C1–C8 (healthy birds), SB1–SB8 (subclinical NE), and CL1–CL8 (clinical NE). Within this biplot, "Loadings" signify the weights of each original variable, while “PC scores” represent the linear combinations of the variables in the dataset

The relative abundance of the jejunal microbial composition was presented at the level of phyla, family, and genus (Fig 4A–C). A total of 16 phyla were identified in jejunum with the phylum Firmicutes the most abundant in birds that developed clinical or subclinical NE, and in CP unchallenged negative control groups. However, a significant increase in the level of Firmicutes was noted in birds that developed clinical (89.1%) and subclinical NE as compared to the negative control group (76%, p < 0.0180) (Fig 4A). The second most abundant phylum was Cyanobacteria which constituted 21.8% in the negative control groups. However, the level of Cyanobacteria was significantly reduced in CP challenged birds that developed clinical (3.21%), or subclinical (11.49%) NE as compared to the unchallenged control group (p < 0.0001). The third most abundant phylum was Proteobacteria which significantly increased in birds with clinical NE (7.54%) as compared to the negative control group (0.44%, p < 0.049). Although the relative abundance of Proteobacteria showed a dramatic increase (0.95% to 4.34%) in birds that developed subclinical NE as compared to the negative control group, it was not statistically significant (p < 0.440) (Fig 4A).

#### Jejunal relative microbial abundance at the family level

At the family level, the negative control group had predominantly *Lactobacillaceae* family (64.3%) followed by *Peptostreptococcaceae* (14.8%), *Oscillatoriophycideae* (13.4%), and other families (7%) (*i.e.,* others represent families with relative abundance of less than 2%) including *Clostridiaceae* (1.46%) and *Enterobacteriaceae* (0.35%) (Fig 4B). Birds with subclinical and clinical NE had a significant decrease in *Lactobacillaceae* and *Oscillatoriophycideae* (p<0.0001) as compared to the CP unchallenged control group. There was significant increase in *Clostridiaceae* in birds with clinical NE (70.4%) followed by subclinical NE (35.3%) as compared to birds not exposed to CP (p<0.0001) (Fig 4B). An increased trend of *Enterobacteriaceae* was observed as CP infected birds develop subclinical NE (3.40%, p<0.048) and transition to clinical NE (6.42%, p<0.046) (Fig 4B).

#### Jejunal relative microbial abundance at the genus level

The genus *Lactobacillu*s (54.9%) was the dominant genus in the family *Lactobacillaceae* (Phylum Firmicutes) in the CP unchallenged control group. In contrast, the genus *Lactobacillu*s was significantly lower in birds with subclinical or clinical NE (p < 0.0001) as compared to the negative control group (Fig. 4C). As expected, the relative abundance of the genus *Clostridium* (Phylum Firmicutes) was significantly higher in birds that developed subclinical (32.74%) or clinical NE (54.89%) as compared to birds not exposed to CP (1.79%, p < 0.0001) (Fig. 4C). Interestingly, the relative abundance of the genus *Romboutsia* (Family *Peptostreptococcaceae*; Phylum Firmicutes) increased in birds that developed subclinical NE (25.57%) as compared to the negative control group (17.73%, p < 0.0323), and significantly reduced in birds that developed clinical NE (16.57%) as compared to birds that developed subclinical NE (p < 0.014). In contrast, there was an increasing trend of the relative abundance of the genus *Escherichia* (family *Enterobacteriaceae,* phylum Proteobacteria) as birds develop clinical NE. The proportion of genus *Escherichia* rose to 4.14% in birds that developed subclinical NE as compared to the CP unchallenged control group (0.31%, p < 0.451). The number of birds that developed clinical NE (8.82%) was statistically significant (p < 0.0216) (Fig. 4C). Based on principal component analysis, the *Lactobacillaceae, Enterobacteriaceae* and *Clostridiaceae* families had distinct clustering between the CP unchallenged control group and birds that developed subclinical or clinical NE (Fig. 4D)**.**

#### Alpha and beta-jejunal microbial diversity of sub-clinical and clinical NE

**Fig. 5:**
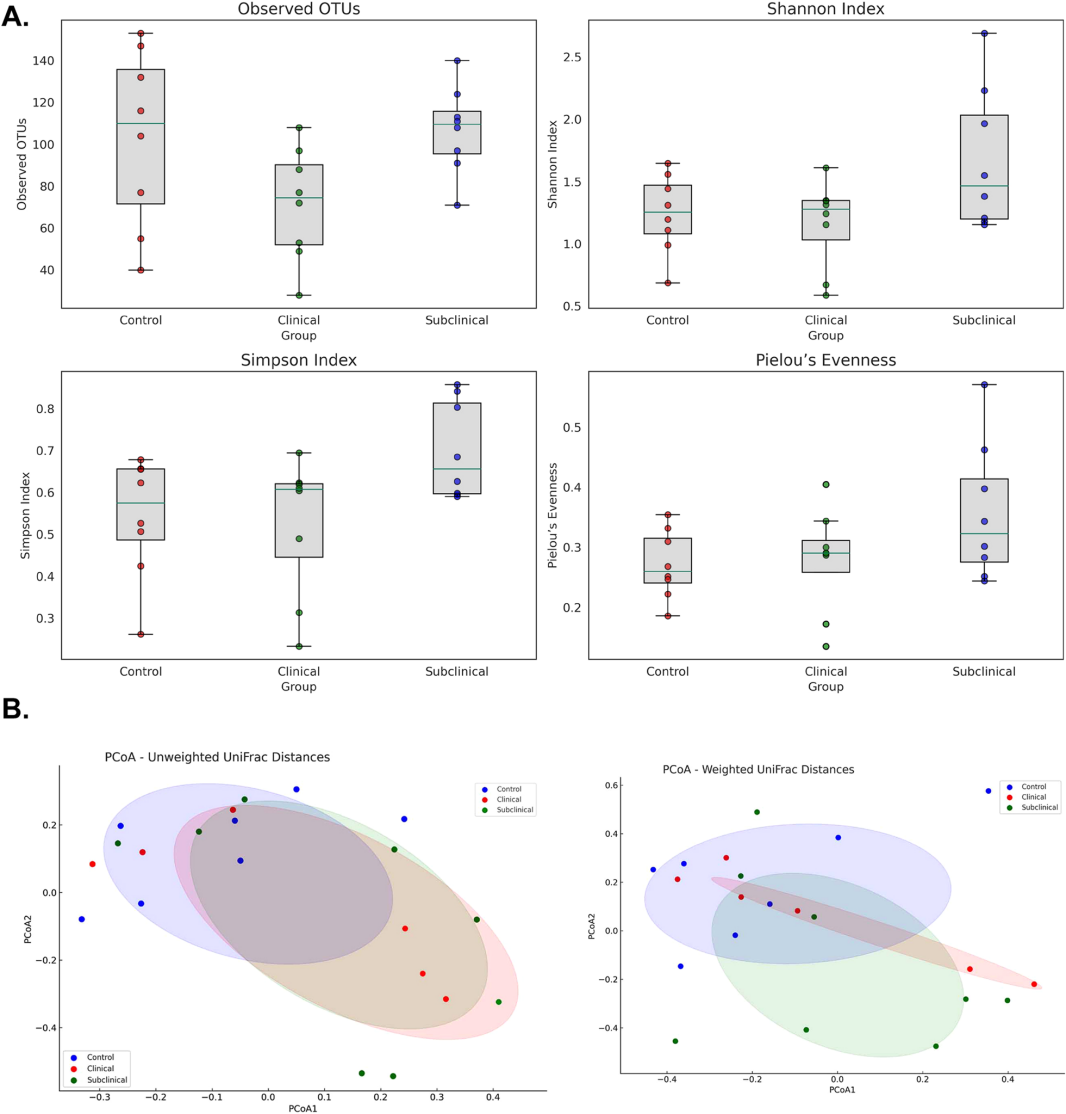
Jejunal microbiome analysis in control and NE-affected birds post-CP infection. **A** Alpha diversity indices of the jejunal microbiome with box plots indicating distribution across control and NE-affected groups (n = 8/group). **B** PCoA plots, based on unweighted and weighted UniFrac distances, illustrating microbial community compositions in the same groups

The microbial diversity within each sample (alpha diversity) of the control birds, birds with subclinical or clinical NE post CP infection was examined by computing richness (observed operational taxonomic units, OTUs), Shannon index, Simpson index and Pielou’s evenness. The box plots generated to visualize the distribution of each metric across the groups displayed a clear distinction in the microbial diversity within the groups (Fig. 5A). The differences in microbial composition between the control, subclinical NE and clinical NE group was assessed by beta diversity analysis by measuring the weighted and unweighted UniFrac distances which were visualized by Principal Co-ordinate Analysis (PCoA) (Fig. 5B). Unweighted UniFrac distances demonstrated statistically significant differences in microbial community structures between the control, subclinical NE and clinical NE groups (p < 0.007; R^2^ = 0.1226; F-value = 3.4001). However, when considering taxa abundance (weighted UniFrac distances), the differences between the groups were not statistically significant (p < 0.229). The variations in the microbial communities are somewhat influenced by the severity of NE, more notably in the unweighted analysis.

### Severity of NE associated with CP isolates containing different combinations of toxin genes

#### Gross and microscopic lesions

**Fig. 6:**
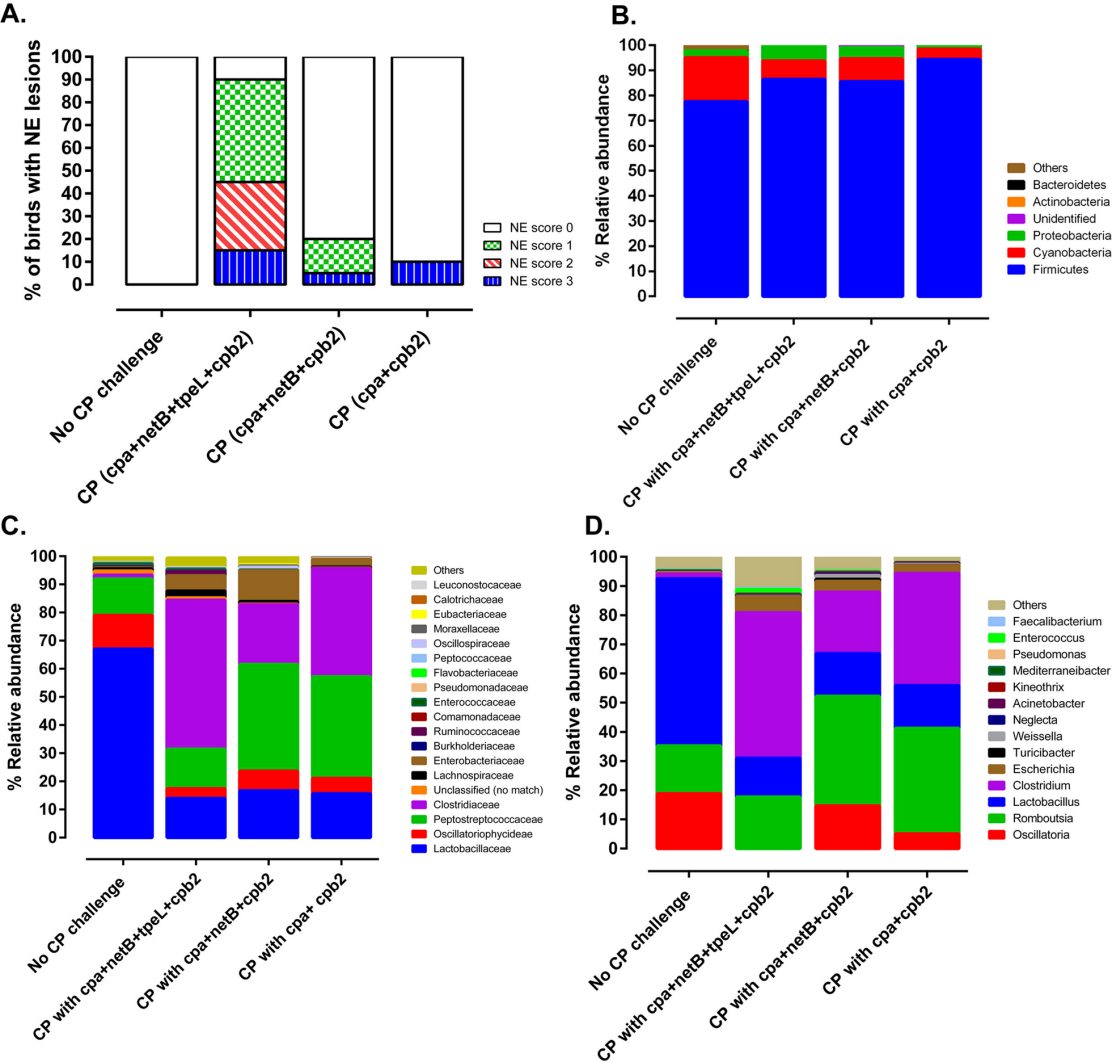
Impact of different CP Toxin gene combinations on NE histologic lesions and jejunal microbiome composition in broiler chickens. **A** Percentage of broiler chickens (n = 20/group) manifesting NE histopathological lesions following exposure to different CP toxin gene combinations (p = 0.0001). **B** Relative abundance (n = 20/group, n = 10 for control) of the jejunal microbiome at the phylum level post-CP exposure. **C** Composition of the jejunal microbiome at the family level. **D** Detailed breakdown at the genus level of the jejunal microbiome. CP: *Clostridium perfringens*

The association of the presence of different combinations of toxin genes in CP isolates and the severity of NE in infected birds was evaluated in the CP broiler chicken infection model. Group 2 (infected with CP containing *cpa*, *netB*, *cpb2* and *tpeL* genes), Group 3 (infected with CP containing *cpa*, *cpb2* and *netB* genes); and Group 4 (infected with CP containing *cpa* and *cpb2* genes) had gross NE lesions at the rate of 15%, 5% and 10%, respectively. No gross lesions were noted in group 1 (unchallenged control group). Groups 2, 3, and 4 had histopathological lesions of NE at the rate of 90%, 20% and 10%, respectively. No histopathologic lesions were observed in the unchallenged negative control group (Fig. 6A).

#### Jejunal relative microbial abundance at the phylum level

The jejunal microbial diversity was presented as relative abundance at the phyla, family, and genus levels (Fig 6B–D). A total of 16 phyla were identified. Like the first experiment, the phylum Firmicutes was the most abundant in all the experimental groups. However, the proportion of Firmicutes increased in birds exposed to *cpa*+*netB*+*tpeL*+*cpb2* (86.45%, p < 0.0017), *cpa*+*netB* +*cpb2* (85.55%, p = 0.0053), or *cpa*+*cpb2* (94.34%, p < 0.0001) as compared to unchallenged control birds (77.52%) (Fig 6B). Cyanobacteria was the second most abundant phylum with its proportion significantly reduced in all challenged groups as compared to the unchallenged negative control group (p < 0.0001). The third most abundant phylum was Proteobacteria whose proportion was 0.95% in the unchallenged control group. In contrast, the abundance of Proteobacteria increased in birds exposed to *cpa*+*netB*+*tpeL*+*cpb2* (5.94%), *cpa*+*netB*+*cpb2* (4.50%), or *cpa*+*cpb2* (1.23%) (p < 0.2960, Fig 6B).

#### Jejunal relative microbial abundance at the family level

Microbial abundance at the family level in the unchallenged negative control group was dominated by *Lactobacillaceae* (61.99%) followed by *Peptostreptococcaceae* (16.02%), *Oscillatoriophycideae* (10.78%) and other families (5.40%) (i.e., including *Clostridiaceae* [1.21%] and *Enterobacteriaceae* [0.31%]) (Fig 6C). Birds exposed to CP isolates containing *cpa*+*netB*+*tpeL*+*cpb2*, *cpa*+*netB*+*cpb2* and *cpa*+*cpb2* had a significant decrease in *Lactobacillaceae* (17.42%, 18.37%, 13.02%, respectively) as compared to the unchallenged control group (p < 0.0001). Similarly, birds exposed to *cpa*+*netB*+*tpeL*+*cpb2*, *cpa*+*netB*+*cpb2* and *cpa*+*cpb2* had a decreased proportion of *Oscillatoriophycideae* (i.e., 3.10%, 7.23%, and 3.87%, respectively) compared to the unchallenged control group (p<0.3250). There was a significant increase in *Clostridiaceae* in birds exposed to *cpa*+*netB*+*tpeL*+*cpb2* (p < 0.0001) compared to the unchallenged control group. An increase in the proportion of *Clostridiaceae* was also noted in *cpa*+*netB*+*cpb2* (p = 0.3540) and *cpa*+*cpb2* (p = 0.4400) compared to the unchallenged negative control group. Similarly, a significant increase in the abundance of *Peptostreptococcaceae* was observed in birds exposed to *cpa*+*netB*+*cpb2* (54.32%) and *cpa*+*cpb2* (71.80%) compared to birds not exposed to CP (16.02%, p < 0.0001) and in birds challenged with CP containing *cpa*+*netB*+*tpeL*+*cpb2* (11.70%, p < 0.774) (Fig 6C).

#### Jejunal relative microbial abundance at the genus level

At the genus level, *Lactobacillus*, *Oscillatoria*, *Romboutsia*, *Clostridium*, *Escherichia* and *Weissella* were identified with a relative abundance of 57.31%, 18.81%, 16.40%, 1.55%, 0.38% and 0.16%, respectively in the unchallenged control group (Fig. 6D). In contrast, there was a significant increase in *Clostridium* (49.88%) in birds challenged with CP containing *cpa* + *netB* + *tpeL* + *cpb2* compared to the negative control group (p < 0.0001). In the same group, there was an increase in the proportion of *Escherichia* (5.34%), *Romboutsia* (17.67%) and *Weissella* (0.23%), and a significant decrease in *Lactobacillus* (13.28%, p < 0.0001). The genus *Oscillatoria* was undetectable in the group. The birds exposed to the CP isolate containing *cpa* + *netB* + *cpb2* genes had a significantly increased proportion of *Romboutsia* (53.32%) and a significantly reduced level of *Lactobacillus* (16.18%, p < 0.0001) as compared to the other groups (p < 0.0001). A rise in the proportion of *Oscillatoria* (14.62%, p < 0.4690), *Clostridium* (8.52%, p < 0.0760) and *Escherichia* (3.49%, p = 0.7050) were also observed. In addition, birds exposed to CP isolate containing *cpa* + *cpb2* had *Romboutsia* (71.23%), *Lactobacillus* (11.79%), *Clostridium* (7.88%), *Oscillatoria* (3.30%), and *Escherichia* (0.96%) (Fig. 6D).

#### Alpha and beta-jejunal microbial diversity based on CP toxin genes

**Fig. 7:**
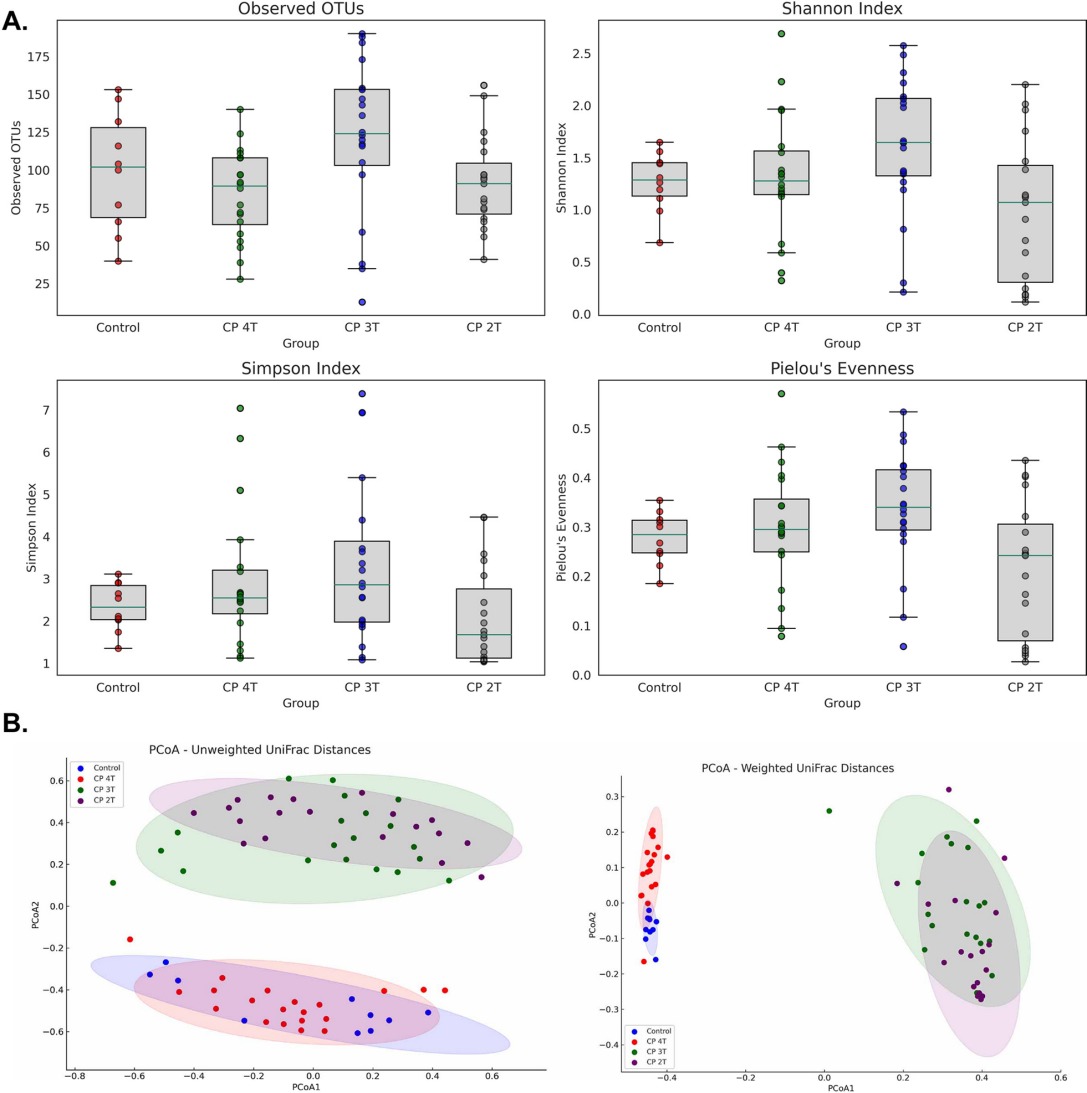
Jejunal microbiome diversity and composition in control and CP toxin-exposed broiler chickens.** A** Alpha diversity indices with box plots showing distribution across control and different CP toxin combinations infected groups (n = 20/group, n = 10 for control). **B** PCoA plots of microbial community compositions, using unweighted and weighted UniFrac distances. Toxin labels: CP 4T includes *cpa*, *netB*, *cpb2*, *tpeL* genes; CP 3T has *cpa*, *cpb2*, *netB* genes; CP 2T contains *cpa *and *cpb2* genes

The microbial diversity within each sample (alpha diversity) of the group of birds infected with CP containing *cpa*, *netB*, *cpb2* and *tpeL* genes (CP 4T), *cpa*, *cpb2* and *netB* genes (CP 3T), *cpa* and *cpb2* genes (CP 2T) or non-infected control birds was examined by computing richness (observed OTUs), Shannon index, Simpson index and Pielou’s evenness. Based on the distribution of each metric across the groups, a clear distinction in the microbial diversity within the groups (Fig. 7A) was observed. The differences in microbial composition between the CP 4T, CP 3T, CP 2T and non-infected control groups was evaluated by beta diversity analysis. The measurement of the weighted and unweighted UniFrac distances was visualized by PCoA **(**Fig. 7B**)**. Unweighted UniFrac distances demonstrated statistically significant differences in microbial community structures between the CP 4T, CP 3T, CP 2T and non-infected control groups (p < 0.001; R^2^ = 0.4932; F-value = 21.085). In addition, weighted UniFrac distances (taxa abundance), between the CP 4T, CP 3T, CP 2T and non-infected control groups were statistically significant (p < 0.001; R^2^ = 0.81816; F-value = 97.485). The high R^2^ values suggest that the toxin types in CP isolates are significant contributors to the observed variations in the microbial communities.

### Differential microbial abundance in NE affected birds.

**Fig. 8:**
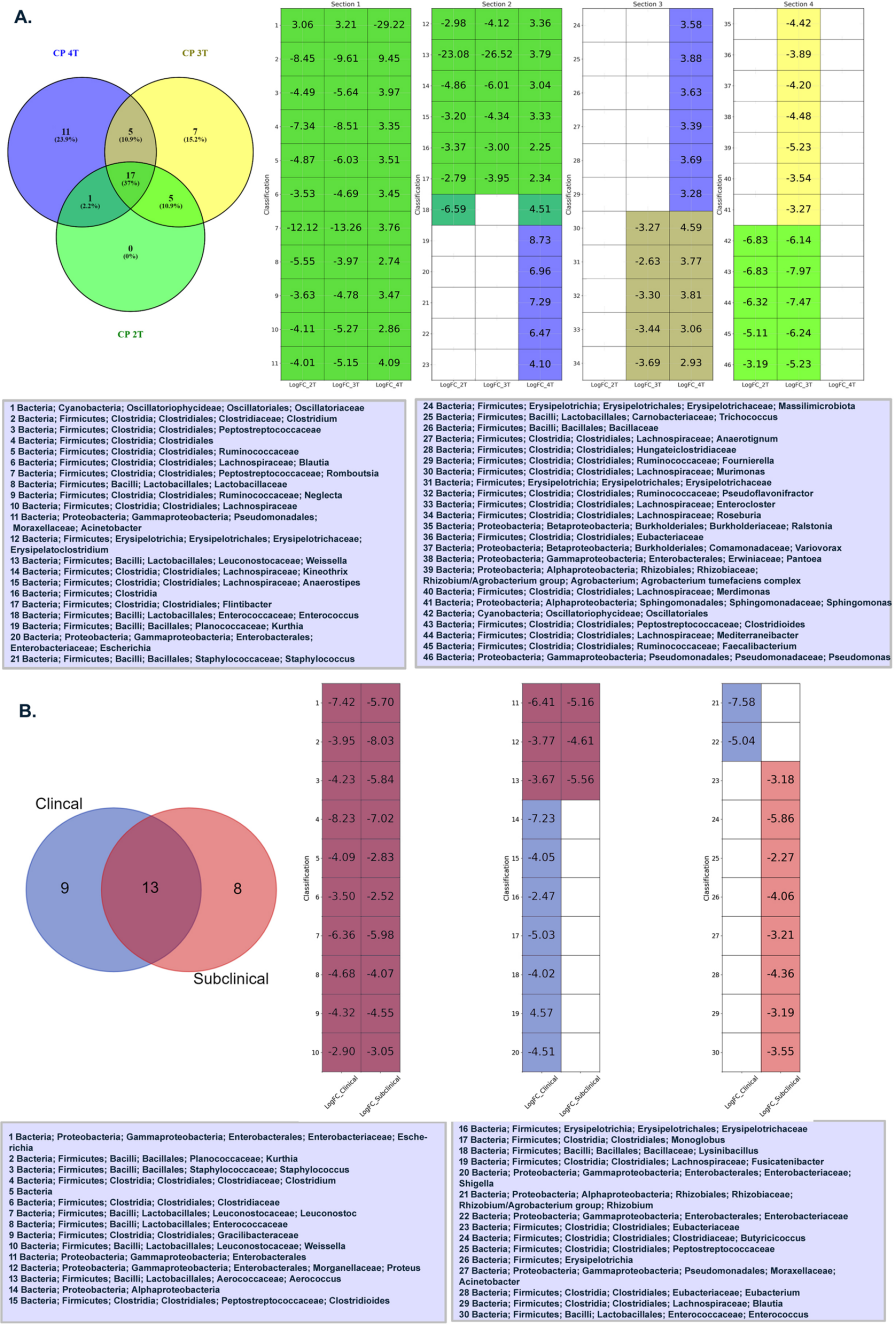
Venn diagram illustrating differential abundance values in the microbiome of broiler chickens. This figure displays a Venn diagram heatmap showing LogFC values of microbiome abundance for five conditions: **A** “CP 4T”, “CP 3T”, “CP 2T”, and **B** “Clinical” and “Subclinical” conditions. Each cell in the heatmap represents a LogFC value, with non-significant values in white. The x-axis represents LogFC value in the clinical conditions, and the y-axis represents microbiome classifications

DESeq2 results have discerned distinct bacterial abundance patterns tailored to specific toxin combinations. The Additional file 2: Fig. S1 and Additional file 3: Fig. S2 provide the statistical analysis of microbial abundance across different comparison groups. The 4T group was characterized by an overabundance of taxa such as *Kurthia*, *Escherichia*, and *Staphylococcus*, primarily within the Firmicutes and Proteobacteria phyla (p < 0.01) (Fig. 8A). In contrast, the 3T group exhibited a decreased abundance of bacterial classifications like *Ralstonia*, *Eubacteriaceae*, and *Variovorax*, mainly from the Proteobacteria and Firmicutes phyla (p < 0.01). Notably, bacterial taxa including *Oscillatoriaceae*, *Clostridium*, and *Peptostreptococcaceae*, primarily from the Cyanobacteria and Firmicutes phyla, maintained consistent abundance across all toxin combinations, inclusive of the 2T group (p ≤ 0.01).

Further dissecting the clinical manifestations of NE, profound variations in bacterial abundance emerged. Birds with clinically severe symptoms showed a decline in several bacteria from the "Firmicutes" phylum, such as *Clostridioides* and *Lysinibacillus*, and also from the "Proteobacteria" phylum, like *Shigella* and *Rhizobium* (p < 0.01), (Fig. 8B). Conversely, birds manifesting subclinical conditions exhibited reduced abundances of bacteria primarily from the "Firmicutes" phylum (p < 0.01). Specifically, among the clinically severe birds, there was an elevated abundance of the ‘*Clostridiales*’ (p < 0.01).

## Discussion

NE is a complex disease and a variety of predisposing factors like *Eimeria* sp. infections, immunosuppressive viral diseases such as IBDV, and poor management factors such as high stocking density, abrupt change in chicken feed ingredients and poor litter quality play a critical role in the occurrence and severity of the disease [34–36]. The degree of virulence of CP also plays a major role in the pathogenesis of NE. CP isolates expressing the plasmid encoded *netB* toxin have been associated with cases of NE [37], and the *netB* gene is infrequently detected in CP isolated from healthy chickens compared to CP isolates linked to NE outbreaks [18, 27, 38]. In addition, CP isolates expressing *tpeL* toxin (large clostridial toxin [LCT] family) [39] along with *netB* toxin produce a more severe form of NE [20]. Apart from *netB* and *tpeL* toxins, the role other CP toxins play in the pathogenesis of the NE is not well understood. Here, we investigated the potential interdependece of *cpa, netB, cbp2* and *tpeL* toxins produced by CP in the pathogenesis of NE. To best of our knowledge it is the first study demonstrating the microbiome analysis based on toxin genes of different CP isolates in broiler chickens and their role in the development of NE. We aslo examined the jejunal microbial dysbiosis associated with subclinical and clinical NE induced by CP isolates containing dfferent combination of toxin genes.

Out of the 57 clinical isolates of CP characterized under this study, the majority contained *cpa* and *netB* toxin genes followed by CPA as a major toxin. A small minority of CP isolates contained *tpeL* gene. This is in line with previous reports indicating that NE outbreaks are mostly associated with CP expressing *netB* toxin [18, 27, 38]. Under this project, we successfully developed a subclinical and/or clinical NE broiler chicken animal model based on vIBDV-SK09 [40] as an immunosuppresant and/or by abrupt increase in the proportion of plant based protein in the chicken diet. In the past, *Eimeria* sp. infection and fish meal based NE animal models have been reported [31, 32, 41, 42]. Since *in vitro* growth and maintainance of *Eimeria* sp. is difficult and time consuming, we believe that our animal model is an easy fit for studying the pathogenesis of NE. Our results demonstrated that immunosupression with vIBDV-SK09 follwowed by CP infection produced increased mortality and severe gross and histolopathologic lesions suggesting immunosuppression as one of the most improtant predisposing factors to NE development. In addition, an abrupt increase in the concentration of protein in the broiler feed effectively precipiateted NE in CP exposed chickens. High concentration of protein in the feed tend to induce CP to express an increased amount of *cpa* toxin. Since CP lacks enzymes for amino acid biosynthesis, high protein diet also acts as a source of amino acids and a substrate for the proliferation of CP [5, 43].

In our animal model, both subclinical and clinical NE were consistently observed in the jejunum. Therefore, our microbiome based microbial diversity study was focused on jejunal samples collected from healthy, subclinical or clinical NE cases. In contrast, most past microbiome studies were focused on the cecum despite the fact that NE lesions occur in the small intestine [31, 32, 44, 45]. These studies also used exposure of birds to *Eimeria* species as a predisposing factor which could directly cause pathologic lesions in the intestine. To the best of our knowledge, this is the first study which demonstrated changes in the patterns of the jejunal microbiome at the different stages of NE development post CP infection. In this study, *Lactobacillus* (57.11%), *Oscillatoria* (18.81%), *Romboutsia* (16.40%) and *Clostridium* (1.35%) were identified as the common bacterial genera consistently identified in the jejunum of healthy broiler chickens. The dominance of *Lactobacillus* in the jejunal microbial community of healthy chickens was also reported in previous studies [46]. Birds that developed subclinical or clinical NE after exposure to CP containing different combinations of toxin genes had significantly reduced proportion of *Lactobacillus* and significantly increased level of *Clostridium.* Similar phenomenon were reported in the ileum [47] and jejunum [46] of birds after experimental induction of NE. The competitive exclusion of *Lactobacillus* might be essential for CP’s successful colonization of the jejunum and induction of the development of NE. The genus *Lactobacillus* is known for its probiotic properties and maintain intestinal health by regulating the intestinal microbiota [48] and by modulating the immune system [49] *Lactobacillus* can also produce bacteriostatic bacteriocin-like compound against pathogenic bacteria [50]. Moreover, the bacteria produces lactic acid upon fermentation of carbohydrates and reduces the intestinal pH making an inhabitable environment for acid sensitive pathogens [51, 52]. Hence, suppression of *Lactobacillus* was considered beneficial for the colonization and growth of enteric pathogens [46]. Reduction in the proportion of other lactic acid producing bacteria like the genus *Weissella* were reported in the jejunum and ceca of birds that developed NE [32, 46]. Interestingly, the level of *E. coli* in the jejunum increased dramatically in the subclinical NE cases as compared to the unchallenged group. The proportion of the genus *Escherichia* continued to rise significantly as the CP challenged birds developed clinical NE which are consistent with previous reports [53, 54]. Similar enrichement of *Escherichia coli* was also reported in the ileum and cecum of mice after infection with porcine CP [55]. It was proposed that *E. coli* may counteract the serum endotoxin secreted by CP [56]. *E.* coli and CP might also have a synergestic effect in the pathogenesis of NE. But further studies are required to clearly elucidate the role *E. coli* plays in the development of NE after CP infection.

In addition, our study indicates that the change in the microbial diversity and reduction in the proportion of *Lactobacillus* sp. was dependent upon the CP toxins. The presence of *netB* and *tpeL* toxin genes caused the most dysbiosis as compared to a combination of *cpa* and *cpb2* toxin genes alone. A distinct pattern in the jejunal microbial population was observed between unchallenged negative control birds and birds that developed subclinical, or clinical NE after challenge with CP containing *cpa*+*netB*+*tpeL*+*cpb2* genes. This indicates that dysbiosis of the normal intestinal microbial community is an essential step in the pathogenesis of NE. Only a few broilers exposed to CP isolates containing either *cpa*+*netB*+*cpb2* or *cpa*+*cpb2* developed NE. Interestingly, birds which did not develop either subclinical or clinical NE had a change in the jejunal microbial community as compared to the negative control group (data not shown). More specifically, these birds had a statstically significant increase in the abundance of the genus *Rombutsia* as compared to the challeneged groups and the unchallenged control groups. This suggests that the genus *Rombutsia* may contribute to the inhibition of the proliferaiton of CP and progression of the infection to subclinical or clinical NE. Previous studies allude that *Rombutsia* metabolizes monosaccharides and disacharides and produces acetic, lactic and fomic acids [57]. Buffered formic acid have shown to improve the the performace of broiler chickens with improved feed conversion ratio [58]. Likewise, supplementation of *Astragalus* polysaccharide to NE affected birds led to the proliferation of *Romboutsia* in the ileum and was correlated with a decreased abundance of CP in the cecum [59]. Altogether, the genus *Rombutsia* might play a significant role in the competitive exclusion of CP and helps in the prevention/control of NE during exposure to CP containing *cpa*+*netB*+*cpb2* or *cpa*+*cpb2* genes. Our results provide evidence that infection of birds with CP containing *cpa*+*netB*+*tpeL*+*cpb2* genes can effectively dampen the proliferation *Romboutsia* and *Lactobacillus* and produce NE.

Beta diversity analyses, comparing microbial composition across groups, revealed significant variations linked to NE severity, influenced by toxin gene combinations. CP 3T (*cpa, cpb2, netB* genes) and CP 2T (*cpa* and *cpb2* genes) exhibit unique beta diversity patterns, shedding light on the interplay between bacterial composition, toxin genes, and NE development in broiler chickens.

Differential abundance analysis of the microbiome in broiler chickens unveils the intricate microbial dynamics underlying disease pathology. In this study, DESeq analysis revealed a stable presence of certain bacterial groups across varying toxin combinations may indicate their inherent resilience or pivotal role in the context of NE in chickens. Intriguingly, both clinical conditions showcased decreased abundance of certain bacteria from the "Firmicutes" and "Proteobacteria" phyla, albeit in distinct ratios. This unveils the nuanced microbial shifts associated with varied clinical presentations of NE, deepening our understanding of the disease's pathology. Collectively, these findings emphasize the intricate dynamics between bacterial abundance and its potential implications in shaping the progression and pathogenesis of NE.

One of the limitations of this study is that the microbiome study was based on 16S rRNA gene sequencing and the microbial classififcation was only possible upto the genus level. Whole genome metagenomic studies will need to be performed in the future for microbiome analysis at the species level. The significance of the chicken intestinal mycobiome and virome in the pathogenesis of NE should also be addressed in the future. Our study also focused on the jejunum as it is the prinicpal site for NE development. But the impact of NE in the microbial community present in the different parts of the small and large intestine needs to be investigated. As *E. coli* was found in high proportions in CP infected birds which developed subclinical or clinical NE, the role *E. coli* plays in the pathogenesis of NE requires further investigation. In addition, the specific *Rombutsia* and *Lactobacillus* species which maintain the health of the jejunum of birds should be identified and tested as potential probiotics for the control and prevention of NE.

In summary, our first objective successfully established a broiler chicken model for NE through an abrupt increase in plant-based protein in the diet and the induction of immunosuppression via vIBDV-SK09 infection. CP isolates carrying the *cpa*+*netB*+*tpeL*+*cpb2* genes induced microscopic lesions. Our second objective consistently revealed distinct differences in the jejunal microbial community between CP-infected birds and healthy ones, serving as a potential marker for identifying subclinical and clinical NE in broiler chickens. The proliferation of *E. coli* in the jejunum of CP-infected birds may play a crucial role in exacerbating NE pathogenesis, although further confirmation is needed. Additionally, our third objective indicates the importance of *netB* and *tpeL* genes in NE pathogenesis, with up to 90% of CP-infected birds carrying *cpa*+*netB*+*tpeL*+*cpb2* genes developing microscopic lesions in the jejunum and significantly impacting jejunal microbial diversity. As the poultry industry moves away from prophylactic antimicrobial use, the resurgence of NE is a growing concern. Our study provides evidence that the *Romboutsia* and *Lactobacillus* genera could be promising candidates for developing probiotic products as effective alternatives for preventing and controlling NE.

## Materials and methods

### CP isolates, and virotyping by polymerase chain reaction (PCR)

Fifty-seven CP isolates were characterized by polymerase chain reaction (PCR) for the presence of toxin genes. The 50 isolates were kindly provided by Dr. Patrick Boerlin, Department of Pathobiology, Ontario Veterinary College (OVC), University of Guelph, Canada. The remaining isolates were provided by Prairie Diagnostic Services (PDS) Inc., University of Saskatchewan. All the CP were isolated from field cases of NE in chickens across the provinces of Alberta, Saskatchewan and Ontario (unpublished). Each CP isolate was individually cultured in 3 mL of brain heart infusion (BHI) broth incubated at 37 °C for 18 hr under anaerobic conditions. Total genomic DNA was extracted using DNeasy Blood and Tissue kit (Qiagen) according to the manufacturer’s instructions. The quality and quantity of the extracted genomic DNA was determined by using the NanoDrop™ spectrophotometer (Thermo Scientific™). The targeted toxin genes of CP (i.e., major toxin genes; including *cpa*, *cpb*, *etx* and *iap*, as well as minor toxin genes including *cpe*, *netB*, *cpb2* and *tpeL*) were examined by PCR as previously described [60]. Briefly, all the PCR reactions were carried out in a final reaction volume of 50 µL using a template DNA of 100 ng and 2 units of Taq DNA polymerase (Thermo Fisher Scientific). The PCR program consisted of 35 cycles of denaturation (95°C for 30 sec), annealing (To varied depending on the gene amplified and performed for 30 sec), and elongation (72°C for 1 min). The primer sequences, annealing T^o^ and fragment size of each toxin gene amplicon is given in Additional file 1: Table S1. Reference CP strains (ATCC 13124, *Clostridium perfringens*; Strain S 107, contain *cpa* gene; ATCC 3626, *Clostridium perfringens*; type b, contains *cpa*, *cpb*, *etx*, *cpb2*, and *pfoA *genes and an in house CP 21 strains which contains *cpa*, *netB*, *cpb2*, and *tpeL* genes) were included as controls. The amplified PCR products were analyzed by gel electrophoresis.

### Preparation of CP for broiler chicken challenge

Clinical isolates of CP containing *cpa*, *netB*, *cpb2* and *tpeL* (toxin type G) toxin genes were selected from our culture collection. To prepare the challenge inoculum, each CP isolate was streaked on separate 5% Columbia sheep blood agar plates in duplicate (Thermo Scientific, Canada) and incubated at 37 ºC for 24 hr, under anaerobic conditions. Single colonies were individually transferred to cooked meat broth media (CMM, Sigma-Aldrich, Canada) and incubated at 37 ºC for 24 hr, under anaerobic conditions. 10 mL of cultured CMM broth was diluted with 490 mL of CMM (dilution factor 50; v/v) and incubated for 24 hr at 37 °C. Subsequently, the CMM culture was used to inoculate fluid thioglycollate media (FTG, Sigma-Aldrich, Canada) (3% [v/v]) and incubated anaerobically at 37 ºC for 15–16 h and 1×10^9^ colony forming units (CFU) per mL was used to challenge broilers at 20, 21 and 22 days of age.

### NE animal model development protocol

Day old broiler chickens (n=120, Fraser Valley Chick Sales, Abbotsford, BC) were placed in the animal care unit (ACU)’s Animal Biosafety level (ABSL)-2 facility, Western College of Veterinary Medicine (WCVM), University of Saskatchewan, Canada. The birds were randomly allocated to 6 groups (n=20/group) as follows: (1) no vIBDV-SK09 and no CP challenge*;* (2) CP challenge in-feed with 20% protein ; (3) CP challenge in-feed with 28% protein; (4) vIBDV-SK09 challenge and CP challenge in-feed with 20% protein; (5) vIBDV-SK09 challenge and CP challenge in-feed with 28% protein; (6) vIBDV-SK09 challenge and CP challenge orally (1 mL of CP culture twice daily for three days) with 28% protein feed. Each group was kept in a separate pen in the same room. Induction of immunosuppression after infection with vIBDV-SK09 followed by an abrupt increase in protein content in the feed was used to predispose broiler chickens to CP infection and NE development. The CP isolate used for this experiment contained *cpa*, *netB, cpb2* and *tpeL* genes. Until 18 days of age, the diet of day-old broiler chickens contained 20% of raised without antibiotics (RWA) poultry starter (Farm Choice^TM^ RWA, MasterFeeds, Canada). Birds were then orally infected with 1x10^3^ TCID_50_/bird (50% tissue culture infectious dose) of vIBDV-SK09 [40] at 17 days of age to induce immunosuppression. vIBDV-SK09 was used since it causes severe immunosuppression by depleting 90% of B-cells in the peripheral blood [61]. Later, feed was withdrawn at 19 days of age for 10-12 hr followed by feeding with 28% RWA feed. The 28% RWA feed was prepared by mixing 25% RWA turkey/gamebird starter crumble (MasterFeeds, Canada, Canada) with 38% layer/grower supplement (MasterFeeds, Canada) at a 10:3 ratio. Broilers were fed with the 28% protein ration with added FTG-grown CP culture in 1:1 (v/w) ratio for 3 consecutive days (20 to 22 days of age). Clinical signs were scored three times per day as follows; 0 = normal; 1= ruffled feathers, reluctant to stand, may peck some feed, may have loose droppings and depressed but still responsive to the environment; 2 = ruffled feathers, sitting posture, hesitates to move, may show brown foamy diarrhea, do not forage, severely depressed; 3 = bird found dead without any pre-monetary signs. Birds were euthanized at 23 days of age and post-mortem examinations were conducted. A diagramatic representation of the experimental procedures is provided (Fig 1). All the animal experiments were approved by the Animal Research Ethics Board, University of Saskatchewan and the experimental procedures were adhered to the Canadian Council on Animal Care guidelines for humane animal use.

### Sample collection protocol for microbiome study

At 23 days of age, the broilers were euthanized, and post-mortem examination conducted. Gross lesions were recorded, and sections of the duodenum, jejunum and ileum were collected for histopathology as described earlier. At the end of the trial, contents of the jejunum (n=8/group) were collected to study the effect of CP challenge on the intestinal microbiome. Samples from broilers with no CP challenge, broilers with macroscopic lesions of NE, and broilers with only microscopic lesions of NE were investigated for the composition of the jejunal microbiome.

### Animal challenge experiments with CP isolates containing different toxin genes

To investigate the role of different toxin combinations in the pathogenesis of NE, challenge experiments were conducted using different CP isolates on the animal-infection model described above. A total of 80 day-old broiler chicks (Prairie Pride, SK, Canada) were randomly divided into four groups (n=20/group): (1) no CP challenge; (2) CP strain containing *cpa*, *netB*, *cpb2* and *tpeL* genes; (3) a CP strain containing *cpa*, *cpb2* and *netB* genes; and (4) CP strain containing *cpa* and *cpb2* genes. The CP culture preparation and animal challenge were similar as mentioned before. Broilers were monitored for development of clinical signs and mortality three times per day until the end of the trial. The experiment was terminated at 3 days post challenge and necropsy was performed. Gross lesions were recorded, and sections of intestines were collected for histopathology. The jejunal contents were collected and stored at -80 °C freezer for DNA extraction and 16S amplicon sequencing.

### Gross and histopathological scoring of the intestine

All dead and euthanized broilers were necropsied (n=20/group) and intestines were carefully opened to record the gross NE lesions. The duodenum, jejunum, and ileum of broilers with and without gross NE lesions were collected in 10% neutral buffered formalin (pH=7.4) for histologic examination. Processing of the fixed intsetinal tissues and hematoxylin and eosin (H & E) staining was performed as described previously [62]. Briefly, fixed intestines were transferred to graded alcohol to xylene solutions for dehydration, followed by clearing. The tissues were then embedded in paraffin wax for sectioning. The tissue sections with a thickness of 3-5 μm were stained with conventional H & E and examined under a light microscope. Microscopic lesions were scored as: 0 = no lesions/ healthy mucosa; 1 = focal necrosis of intestinal villi, acute; 2 = necrosis of intestinal villi, multifocal to coalescing, acute; 3 = diffuse, necrosis of intestinal villi, acute, severe.

### Genomic DNA extraction and 16S amplicon sequencing

Total DNA was extracted from jejunal contents (200 mg/sample) using a QIAamp® Fast DNA Stool Mini kit (Qiagen Inc.). The DNA concentration was measured by a NanoDrop spectrometer (Thermo Scientific™, USA). The 16S amplicon PCR (targeting the V3-4 region) was performed as per the Illumina 16S metagenomic sequencing library preparation protocol. The PCR products were purified using AMPure XP magnetic beads (Beckman Coulter, Inc, CA, USA). The size of the amplicons was confirmed by gel electrophoresis. Sequence libraries were prepared using the Nextera XT DNA Library Preparation Kit (Illumina, San Diego, USA) with 24 or 96 indexes as per the manufacturer’s instructions. The libraries were cleaned up with AMPure XP magnetic beads and analyzed by a bioanalyzer using a high sensitivity DNA kit (Agilent, Santa Clara, CA). The concentration of the amplicon sequence libraries was measured using the Qubit dsDNA BR (broad range) assay kit (Thermo Fisher Scientific, Waltham, USA). The normalized libraries were pooled, denatured with 0.2N NaOH, diluted using H1 hybridization buffer and loaded onto the MiSeq V3 (600 cycles) cartridge. Sequencing was performed using the Illumina MiSeq platform (Illumina, San Diego, US) as per the company’s protocol. The sequence quality was examined in real time using the Sequencing Analysis Viewer (SAV) V1.8.

### Amplicon sequence assembly and taxonomic classification

The FASTQ paired sequence data was extracted from the MiSeq and the data quality metrices were examined by the Illumina SAV V1.8 software. The FASTQ files were then imported into Geneious Prime 2022.01 software (Dotmatics Inc.) and microbiome analysis was performed using the 16S Biodiversity analysis tool. The paired end reads were quality trimmed using BBDuk plugin with the minimum quality score and minimum length set at 30 and 100, respectively. The trimmed sequences were merged using the BBMerge tool. The contigs were then assembled and clustered into operational taxonomic units (OTUs) using the Geneious *de novo* assembler. The representative sequences were BLASTed to a curated 16S rRNA gene reference sequence database from NCBI to assign taxonomy. Finally, the BLAST results were used as a targeted taxonomic database to quantify the biodiversity in the full data set using the Geneious Prime sequence classifier plugin.

### Microbiome diversity and abundance analysis

The jejunal microbial community composition data collated from both data sets were used to perform microbiome diversity analysis. The first dataset contained the relative abundance of microbial taxa for three different groups (n = 8/group; total = 24 samples) which included birds that developed subclinical, and clinical NE post CP infection and non-infected control group. The second dataset contained the relative abundance of jejunal microbial taxa for 4 different groups (20 birds/group except group 1 [n = 10]) including birds which were infected with a CP strain containing *cpa, netB, cpb2* and *tpeL* genes (CP 4 T), a CP strain containing cpa, cpb2 and netB genes (CP 3 T), CP strain containing cpa and cpb2 genes (CP 2 T), and a non-infected control group. To evaluate the diversity within individual samples (alpha diversity), we calculated metrics including richness (observed OTUs), the Shannon index, the Simpson index, and Pielou’s evenness. This analysis was performed using the skbio.diversity.alpha module within the scikit-bio library in Python [63]. The differences in microbial composition between groups (beta diversity) were analyzed by computing the weighted and unweighted UniFrac Distances. These phylogenetic distance metrics were computed using the phyloseq and ape packages in R. Using the computed beta diversity metrices, PCoA was conducted to visualize the differences in microbial community structures among the samples in two-dimensional space. Permutational Multivariate Analysis of Variance (PERMANOVA) was employed to statistically test the differences in microbial compositions between the groups. This was executed using the `adonis2` function from the `vegan` package in R [64]. The test was applied on both unweighted (presence/absence) and weighted (abundance) UniFrac distances. Additionally, DESeq2 analysis was performed to identify taxa that exhibited significant differential abundance between experimental conditions or groups in microbiome datasets derived from sequencing data. This analysis was performed using the DESeq2 package in conjunction with the phyloseq [65] package in the R program.

### Statistical analysis

Survival, gross and histopathological data were analyzed using GraphPad Prism V6.0 software (Dotmatics Inc.) with a significance level of p < 0.05. Survival or mortality data were compared between the groups using the long-rank (Mantel-cox) and chi-square tests, respectively. Microscopic lesions of the intestine were analyzed between the groups using non-parametric Kruskal-Wallis one-way ANOVA and Tukey’s multiple comparison (p < 0.0001). Further comparison of microscopic lesion scores between two groups was analysed with two-tailed Mann Whitney test (p < 0.0001). Family, phylum, and genus level relative microbial diversity were analyzed by Ordinary two-way ANOVA (p < 0.0001). Further, Turkey’s multiple comparison was performed to identify variation between the different experimental groups at 95% confidence interval. Individual and group sample variance at the family level was examined by principal component analysis using GraphPad Prism V6.0 software (Dotmatics Inc.). Alpha diversity metrics across test groups were assessed using the Kruskal-Wallis and Dunn’s post hoc tests. Beta diversity was analyzed using weighted and unweighted UniFrac distances. Differential abundance, based on DESeq2 logFC values (± 1.5 fold), was tested using the Wald test. A p-value of p ≤ 0.01 was considered statistically significant.

### Supplementary Information

**Additional file 1: Table S1.** The primer sequences and the annealing temperature used to amplify CP toxin genes including the expected size of the amplicons.

**Additional file 2: Figure S1.** Differential gene expression in clinical and subclinical conditions. **A** Volcano plots showcase the balance between gene expression magnitude and statistical significance. **B** MA plots illustrate the correlation of average gene expression with log2 fold changes. **C** Box plots highlight the top 5 classifications based on their significant LogFC and p-values.

**Additional file 3: Figure S2**. Detailed analysis of differential gene expression. **A** Volcano plots provide a holistic representation, emphasizing classifications with pronounced differential expressions. **B** MA plots detail the data, showing average expression versus log2 fold changes. **C** Box plots display the top 5 classifications chosen for their pronounced LogFC and compelling p-values.

## Data Availability

The data supporting the findings of this study are available from the authors upon request.
