# Supplementary material for: Exploring the predictive power of jejunal microbiome composition in clinical and subclinical necrotic enteritis caused by Clostridium perfringens: insights from a broiler chicken model
Source: J Transl Med. 2024 Jan 19;22:80. doi: 10.1186/s12967-023-04728-w (PMC10799374; doi:10.1186/s12967-023-04728-w)
Supplement: Supplementary file 1 — Additional file 1: Table S1. The primer sequences and the annealing temperature used to amplify CP toxin genes including the expected size of the amplicons. [file 12967_2023_4728_MOESM1_ESM.docx]

**Table S1.** The primer sequences and the annealing temperature used to amplify CP toxin genes including the expected size of the amplicons.

| Gene | Primers Sequence (5 ́-3́) | Annealing T (°C) | Product (bp) | Reference |
| --- | --- | --- | --- | --- |
| *cpa* | AGTCTACGCTTGGGATGGAA  TTTCCTGGGTTGTCCATTTC | 55 | 900 | [63] |
| *cpb* | TCCTTTCTTGAGGGAGGATAAA  TGAACCTCCTATTTTGTATCCCA | 56 | 911 | [63] |
| *cpb2* | AGATTTTAAATATGATCCTAACC  CAATACCCTTCACCAAATACTC | 53 | 567 | [64] |
| *etx* | TGGGAACTTCGATACAAGCA  TTAACTCATCTCCCATAACTGCAC | 56 | 396 | [63] |
| *iap* | AAACGCATTAAAGCTCACACC  CTGCATAACCTGGAATGGCT | 57 | 293 | [63] |
| *cpe* | GGGGAACCCTCAGTAGTTTCA  ACCAGCTGGATTTGAGTTTAATG | 57 | 506 | [63] |
| *tpeL* | ATATAGAGTCAAGCAGTGGAG GGAATACCACTTGATATACCTG | 55 | 466 | This study |
| *netB* | CGCTTCACATAAAGGTTGGAAGGC  TCCAGCACCAGCAGTTTTTCCT | 61 | 316 | This study |
