# Supplementary figures and images for: Exploring the predictive power of jejunal microbiome composition in clinical and subclinical necrotic enteritis caused by Clostridium perfringens: insights from a broiler chicken model
Source: J Transl Med. 2024 Jan 19;22:80. doi: 10.1186/s12967-023-04728-w (PMC10799374; doi:10.1186/s12967-023-04728-w)

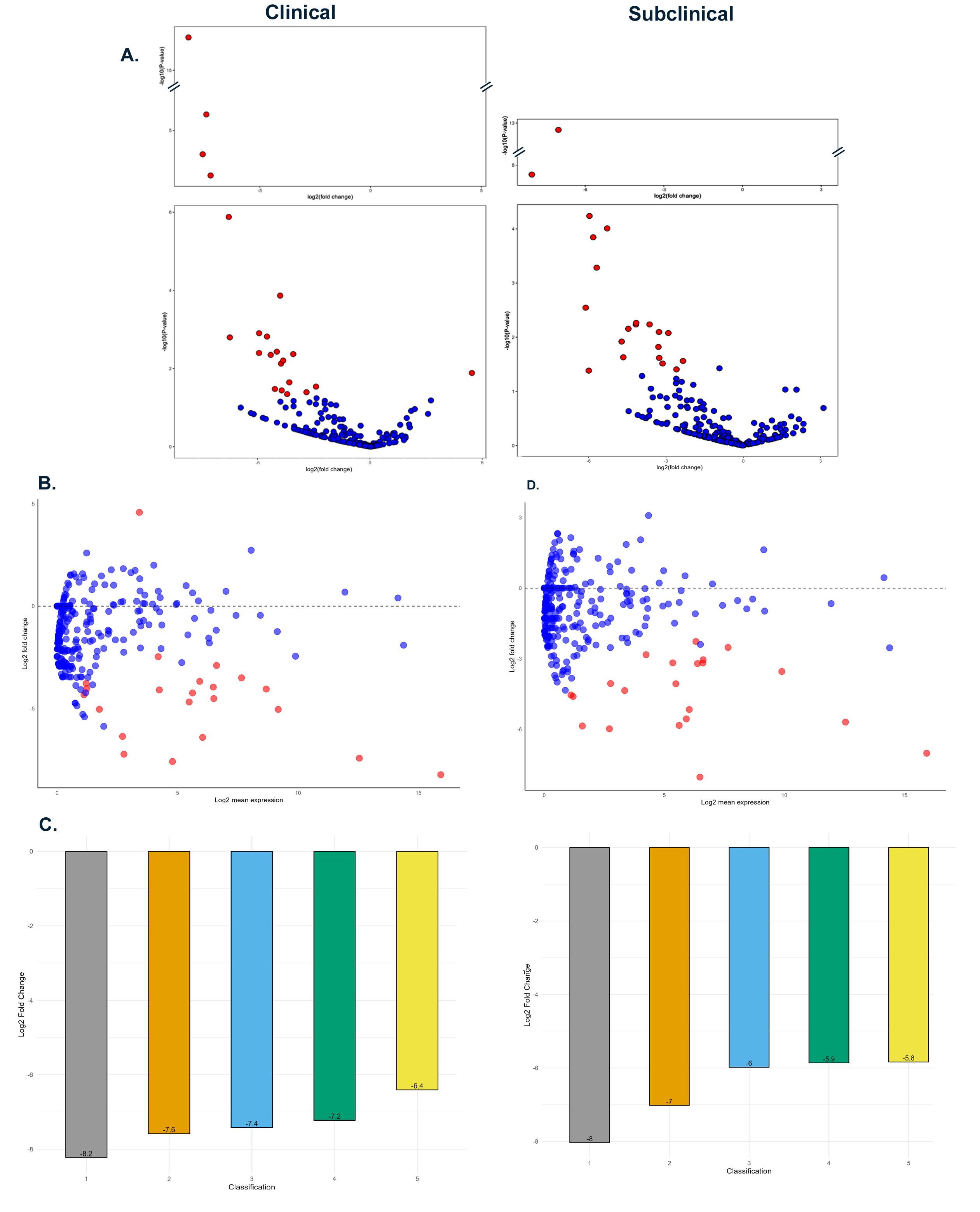

Supplement: Supplementary file 2 — Additional file 2: Figure S1. Differential gene expression in clinical and subclinical conditions. A Volcano plots showcase the balance between gene expression magnitude and statistical significance. B MA plots illustrate the correlation of average gene expression with log2 fold changes. C Box plots highlight the top 5 classifications based on their significant LogFC and p-values. [file 12967_2023_4728_MOESM2_ESM.jpg]

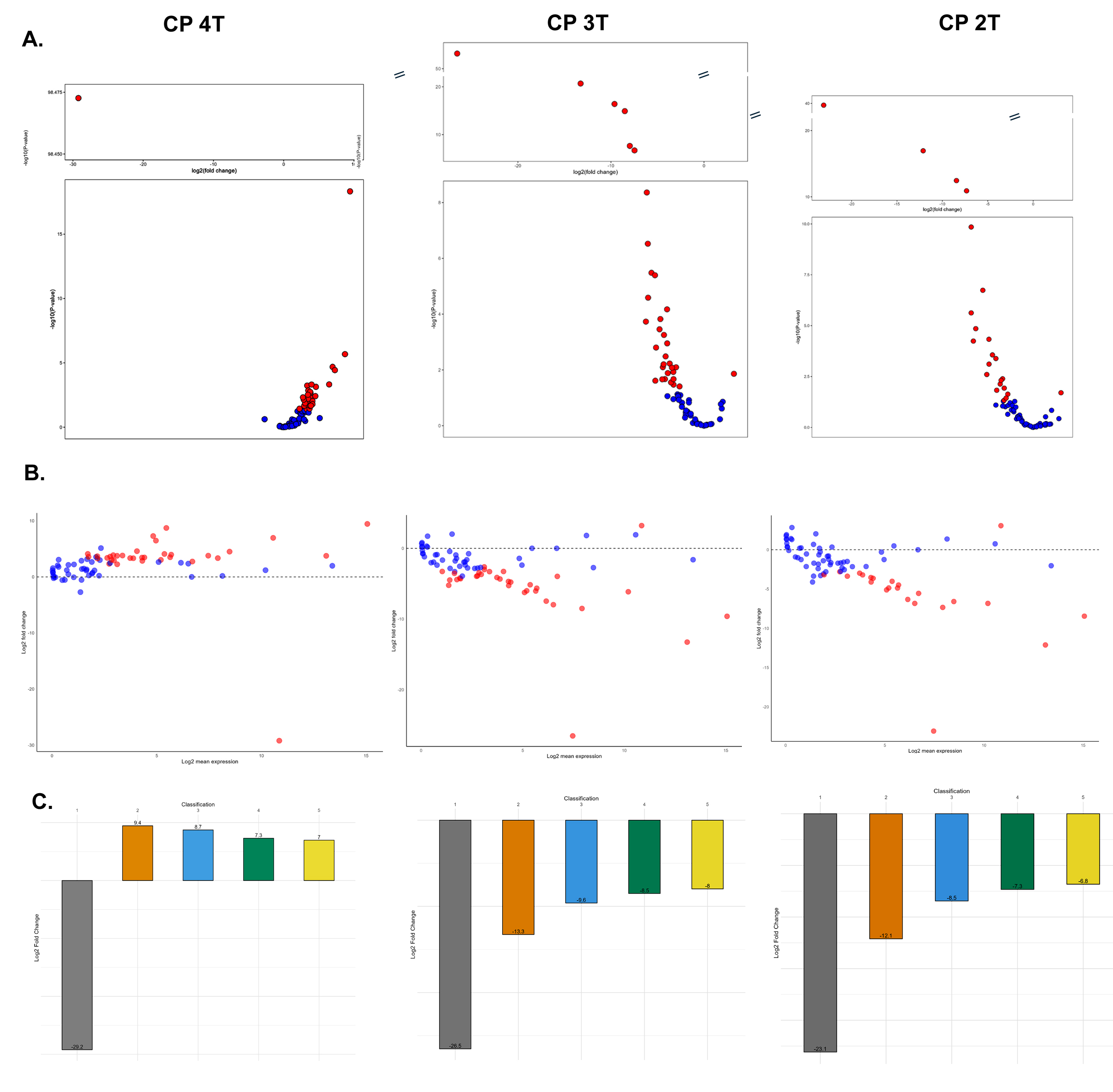

Supplement: Supplementary file 3 — Additional file 3: Figure S2. Detailed analysis of differential gene expression. A Volcano plots provide a holistic representation, emphasizing classifications with pronounced differential expressions. B MA plots detail the data, showing average expression versus log2 fold changes. C Box plots display the top 5 classifications chosen for their pronounced LogFC and compelling p-values. [file 12967_2023_4728_MOESM3_ESM.jpg]
